# Real-time motion force-feedback system with predictive-vision for improving motor accuracy

**DOI:** 10.1038/s41598-024-52811-z

**Published:** 2024-01-25

**Authors:** Ryo Matsui, Tadayoshi Aoyama, Kenji Kato, Yasuhisa Hasegawa

**Affiliations:** 1 The Development of Micro-Nano Mechanical Science and Engineering, Nagoya University, Nagoya, Aichi 464-8603 Japan; 2 Assistive Robot Center, National Center for Geriatrics and Gerontology, Obu, Aichi 474-8511 Japan

**Keywords:** Learning and memory, Motor control

## Abstract

Many haptic guidance systems have been studied over the years; however, most of them have been limited to predefined guidance methods. Calculating guidance according to the operator’s motion is important for efficient human motor adaptation and learning. In this study, we developed a system that haptically provides guidance trajectory by sequential weighting between the operator’s trajectory and the ideal trajectory calculated from a predictive-vision system. We investigated whether motion completion with a predictive-vision system affects human motor accuracy and adaptation in time-constrained goal-directed reaching and ball-hitting tasks through subject experiments. The experiment was conducted with 12 healthy participants, and all participants performed ball-hitting tasks. Half of the participants get forceful guidance from the proposed system in the middle of the experiment. We found that the use of the proposed system improved the operator’s motor performance. Furthermore, we observed a trend in which the improvement in motor performance using this system correlated with that after the washout of this system. These results suggest that the predictive-vision system effectively enhances motor accuracy to the target error in dynamic and time-constrained reaching and hitting tasks and may contribute to facilitating motor learning.

## Introduction

When people exercise, they learn from the results of the motion performance, whether the motion is good or bad. As the learning experience is accumulated, an internal model is created in which appropriate motor commands are accumulated. Human motion becomes more skilled by using the predictions from the internal model^1^. We consider it possible to make the motion proficiencies sequence more efficient by transmitting a robot’s motor control to the human and making it perform better than the normal learning process. Kümmel et al. conducted a study in which pre-defined wrist motion timings were induced in golf novices during a golf swing motion. As a result, they found that the taught motion timing persisted seven days after the guidance and concluded that the initial experience of correct motion by robot guidance may promote motor learning in humans^2^. Crespo et al. also conducted studies in which haptic guidance was provided to humans during the operation of a steerable wheelchair. Their experiments showed that haptic guidance enhanced humans’ ability to learn timing cues in their motion and that the learning effect was more effective in people with lower initial skills^3,4^. These studies considered robot guidance effective for novice learners to learn motor actions.

Conversely, experiments by Crespo et al. did not show much learning effect in humans with high initial skills. The guidance hypothesis that haptic guidance during training can adversely impair motor learning depending on how it is given has long been studied in relation to motor learning^5,6^. Guadagnoli and Lee stated that motor learning is related to information arising from performance and should be optimized according to the learner’s skill level^7^. Most of the previously described haptic guidance experiments are limited to guiding the user to a pre-defined human timing or motion trajectory, which is not varied by the user. Therefore, we considered that calculating the ideal motion from the learner’s motion and transmitting it to the human could improve performance and efficiently promote motor adaptation and motor learning.

For haptic guidance to be achieved in the calculated trajectory by the computer, highly accurate motion of the robot and the ability to calculate the ideal motion in real time are essential. In the field of robot control, improvements in robotics and other technologies have made it possible to drive robotic systems with higher accuracy than in the past. The Da Vinci system is currently an alternative for surgeons to directly operate on patients^8^, and micro-manipulation technology can handle very small objects that humans cannot manipulate^9^. Robotic systems are very valuable because they can be driven with a higher accuracy than that achievable by humans. However, most of them have been limited to those for which the driving trajectory has been pre-defined by humans or for which human manipulation has been input.

With the subsequent development of high-speed image technology, the robot can now be driven independently without relying on human commands. This technology achieves ”predictive-vision,” which is the estimation of the short-distance future of objects observed by acquiring their velocities and accelerations at high speeds. For example, Aoyama et al. applied predictive-vision to a flower stick and developed a high-speed juggling system by estimating the stick’s future position and posture^10,11^. Kim et al. developed a system that can even catch objects with a changing mass distribution by applying ”predictive-vision” to the objects and constantly updating the predicted flight trajectory^12^. These robotic systems plan the driving trajectory of the robot by computer based on the predictive-vision system’s future data, thus realizing human-independent driving. As shown in these examples, robotic systems can now independently be driven with a higher accuracy than humans can achieve. However, most of them have only focused on developing robots and have not worked on transmitting high-accuracy positions and velocities to humans with haptic guidance.

Although there are various possible methods for transferring robot motions to humans by force guidance, direct contact between humans and robots is dangerous. For example, if a human and a robot perform dynamic and large-scale motions in the same space, the rigid robot may collide with the human and cause injury. Therefore, we introduced a teleoperated robot to ensure safety by spatially separating the robot part that performs the task and the part that transmits the motion. The introduction of a haptic device in the operating interface of the teleoperation robot allows the operator to receive force guidance from the system while operating the teleoperation robot.

Based on the above, we hypothesize that robots can facilitate human motor adaptation and motor learning by transmitting to the operator highly accurate motion trajectories designed for the individual operator. In this study, we constructed a system in which the computer simultaneously guides the operator to a guided trajectory weighted between the operator’s trajectory and an ideal trajectory in the simple goal-directed tasks of upper limb reaching and ball-hitting motions. The system was equipped with predictive-vision and controlled with higher position and velocity accuracy than the accuracy of the novice operator’s motion. However, the direct transmission of the computer-calculated dynamic motion of the robotic system to the human body is very risky. Therefore, in this study, a teleoperation robot was introduced to ensure safety by dividing the task-performing robot part and the motion-transmitting part. In this system, a haptic device is introduced as the operating interface of the teleoperation robot so that the operator can receive guidance from the system while operating the robot. Finally, we conducted an experiment on human participants to investigate whether the force guidance can affect human motor learning or adaptation. All participants performed a dynamic goal-directed task using the proposed system, and only half of the participants were provided force guidance from the proposed system in the middle of the experiment to investigate how their motor adaptation changes.

## Methods

### Human-machine system

**Figure 1:**
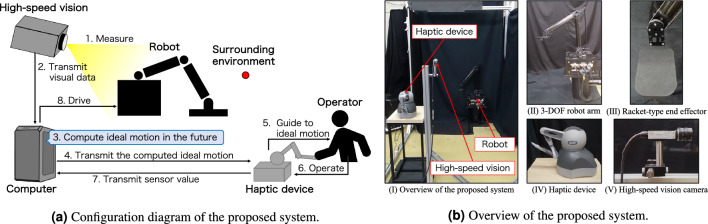
The predictive-vision system proposed in this paper.

Figure 1a shows a configuration diagram of the proposed system. This system integrates a teleoperation robot system with a high-speed vision system and a haptic device. The high-speed vision system measures the velocity and acceleration of the observed objects. Subsequently, the computer calculates the ideal motion of the robot in the future based on these measurements. The haptic device is used as a manipulation device to guide the human hand position to the ideal position and transmits the sensation of ideal motion to the operator forcefully and in real-time. The operator manipulates the haptic device based on the force feedback and indirectly operates the teleoperated robot via the haptic device and the computer. The guidance methods, such as ideal motion and guidance force calculation, are described in the Assist algorithm subsection.

An overview of the system is presented in Fig. 1b. The teleoperation system comprises a 3-degrees of freedom (3-DOF) robot arm, a control PC (Windows 7 Enterprise, 64-bit OS, Intel Core i7, CPU 950, 7.07GHz, DDR3, 12GB), and a haptic device (Touch, 3D systems), a high-speed vision system (IDP-Express R2000, Photron/Hiroshima Univ.). In this study, the robot arm is driven by a brushless DC motor (EC40, Maxon) through harmonic drive gears and operated with only two degrees of freedom based on the tip position data of a human-operated haptic device. The end-effector also has a 10 mm thick plastic attachment with a 130 mm height, 80 mm width, and 20 mm radius rounded rectangular corners, to which a table tennis racket rubber is attached. The high-speed vision system is located 1.25 m from the robot arm, and the frame rate is set to 1000 fps. The robot arm is driven with a motion distance equal to 12.5 times the magnitude of the manipulation input to the haptic device. This magnification factor was determined from the ratio of the motion range of the robot arm to the motion range of the haptic device.

### Experimental procedure

In this study, we investigated whether motion completion with a predictive-vision system affects human motor accuracy and adaptation in time-constrained goal-directed reaching and ball-hitting tasks through subject experiments. All experiments were approved by the Ethics Committee of the Department of Engineering at Nagoya University (21-16). The experiment was conducted after obtaining informed consent from all participants, following the ethical rules for experiments on human subjects at Nagoya University.

**Figure 2:**
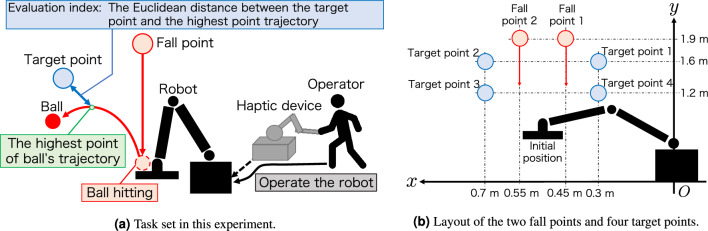
Overview of the task in this experiment. (**a**) The operator manipulates the haptic device to drive the robot. A racket attached to the robot’s end-effector is used to hit back a free-falling ball. The Euclidean error between the highest trajectory point of the ball and the target point is the evaluation index. (**b**) The target point was changed in counterclockwise order, and the fall point was changed appropriately so that all combinations with the target point occurred the same number of times.

All participants performed a task in which they hit a free-falling ball back towards a predefined target point with a table tennis racket attached to a teleoperation robot. The evaluation index was the Euclidean distance between the target point and the highest trajectory point of the returned ball. This is shown in Fig. 2a. To adjust the difficulty level of the task, we used two fall points to start the ball dropping, and four target points were placed in a rectangular shape, as shown in Fig. 2b. The two fall points were set at a height of 1.9 m from the floor, 0.45 m, and 0.55 m from the robot in the horizontal direction. The four target points were set at a height of 1.6 m and 1.2 m from the floor and 0.3 m and 0.7 m horizontally from the robot. All target points and fall points are in the same plane as the two-dimensional plane in which the racket attached to the teleoperation robot arm moves. We have confirmed that each fall point has a plane perpendicular to the gravitational direction with a level. The ball free-falls from the Fall point and moves in the same z-coordinate as the racket. The initial position of the table tennis racket is set 1.0 m vertically downwards from the midpoint of the two fall points.

**Figure 3:**
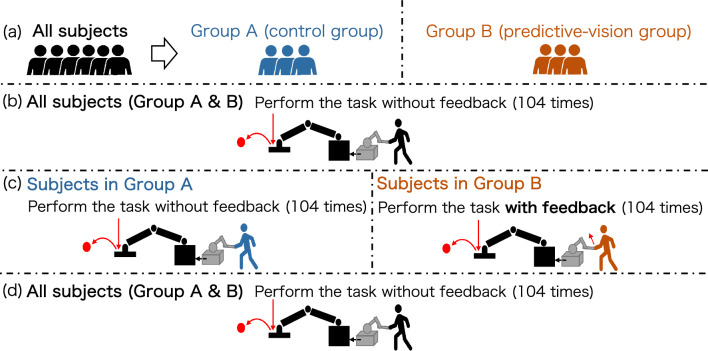
Procedure in this experiment. (**a**) All participants were divided into two groups: Group A (control group) and Group B (predictive-vision group). (**b**) In the first set, all participants in each group performed the hitting task. (**c**) In the second set, only participants in Group B were provided with force feedback, while participants in both groups performed the hitting task. (**d**) In the third set, all participants in each group performed the hitting task.

The experiment procedure is shown in Fig. 3. Twelve healthy participants (4 women and 8 men) were included in this experiment. All participants repeated the task over 312 trials in three sets, 104 trials per set. Even if the highest trajectory point of the ball is not recorded, such as the ball not hitting the racket, the data are discarded, and the trial is not conducted again to keep the number of trials constant for all participants. For each task trial, the target point was changed in counterclockwise order, and the fall point was changed appropriately so that all combinations with the target point occurred the same number of times. After each trial, the subjects were given verbal feedback on the evaluation index, i.e., the Euclidean error between the target point and the highest trajectory point of the returned ball. Before starting the experiment, the participants were divided into Group A (control group) and Group B (predictive-vision group) of six each, and force-feedback instruction was only given to the second set of Group B. In the other trials, no force instruction was given in each group. For the first set, all participants in each group performed the hitting task with their own motion without any intervention. In the second set, only Group B was given force-feedback guidance to test the difference in performance due to the system intervention. Then, in the third set, the participants of Group B were washed out of the guidance. In doing so, we examined the re-adaptation by removing the intervention for participants in Group B.

The evaluation indices obtained in this procedure were averaged over all the participants in each group for each trial, and the learning curve for each set was calculated through an exponential approximation using the trust region method as follows:

1 $$\begin{aligned} E(T) = a \cdot \exp (-bT) + c \cdot \{1 -\exp (-bT)\}, \end{aligned}$$

where *T* is the number of trials; *E*(*T*) is the approximate evaluation index at the *T*th trial; and *a*, *b*, and *c* are the coefficients obtained by the approximation. In this case, *a* represents the intercept of the fitting function, *c* is the convergence value when *T* is set to infinity, and *b* is the convergence speed. The exponential approximation is used because the learning curve considers the transfer function of a first-order linear system that responds to stepwise input actions^13,14^. Furthermore, the degree of improvement in the evaluation index was calculated from the results of the first and third sets using this approximate formula, wherein neither group received any force instructions. It was assumed that the larger the amount of improvement, the more advanced human motor adaptation. Consequently, the changes in motor accuracy with and without the force feedback instruction are discussed.

To determine whether there are statistically significant differences in task performance improvement between sets and groups, we conduct a two-way factorial analysis of variance (ANOVA) with Tukey’s honestly significant difference (HSD) test for multiple comparisons. The performance improvement of each subject from their initial ability is calculated by subtracting the mean error of the second or third set from the mean error of the first set. The ANOVA involves two independent variables: groups and experimental session progressions. The dependent variable is the improvement in performance for each subject in each set. In this experiment, the significance level is set at 0.05, and all tests are conducted using Matlab software.

### Assist algorithm

The proposed predictive-vision system predicts the state of an object in the short-distance future by acquiring its state quantities using a high-speed vision system. In this study, the system applies state prediction to a free-falling ball, calculates the behavior of the ball to hit it back accurately, and provides a force feedback sensation of the motion to the operator.

**Figure 4:**
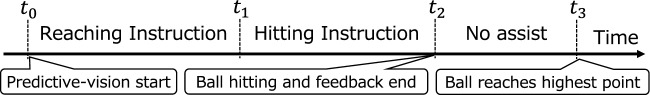
Variable definitions for time are given: $t_0$ indicates the time when predictive-vision started, $t_1$ indicates the time when hitting instruction started, $t_2$ indicates the time of ball hitting, and $t_3$ indicates the time when the ball reached the highest point, respectively.

The algorithms used in this study are divided into two types: (1) an algorithm that calculates the behavior to perform the task ideally by the system using predictive-vision and determines the assistance to be provided to the operator, and (2) an algorithm that instructs the operator based on the determined assistance in a force-feedback manner. The two algorithms are executed in sequence. The force output values are calculated immediately after the ideal behavior is calculated.The former algorithm can be further divided into the reaching instructions, which predict the point where the ball will fall based on future state quantities and contact the ball with the racket, and the hitting instructions, which accurately hit the free-falling ball. In this paper, $t_0$ is the time when predictive-vision starts, $t_1$ is the time when reaching instruction ends and hitting instruction starts, $t_2$ is the time when the ball makes contact with the racket, and $t_3$ is the time when the returned ball reaches the highest point. This is shown briefly in Fig. 4. As shown in this diagram, either one of the two Instructions is adopted depending on time and never at the same time. The gravitational acceleration is denoted by *g*. The x- and y-axes indicate the horizontal and vertical directions, respectively. Detailed descriptions of these algorithms are provided in this section.

#### Ball velocity calculation method

In this study, a free-falling ball was subjected to high-speed image measurements to estimate future state quantities. The positional data on the center of mass of the ball were obtained by binarizing the images captured using high-speed vision at 1000 fps. Subsequently, the noise in the position data was removed by applying a forty-one-point moving average filter to the collected data. By applying this filter, data 20 ms after the image acquisition time is output 40 ms after the image acquisition. In other words, there is an apparent latency of 20 ms in acquiring the ball position information. Afterwards, by applying the corrections described in this section to the filtered data, the translational velocity of the ball, $v_b = [v_{bx}, v_{by}]^T$, is calculated every 1 ms.

**Figure 5:**
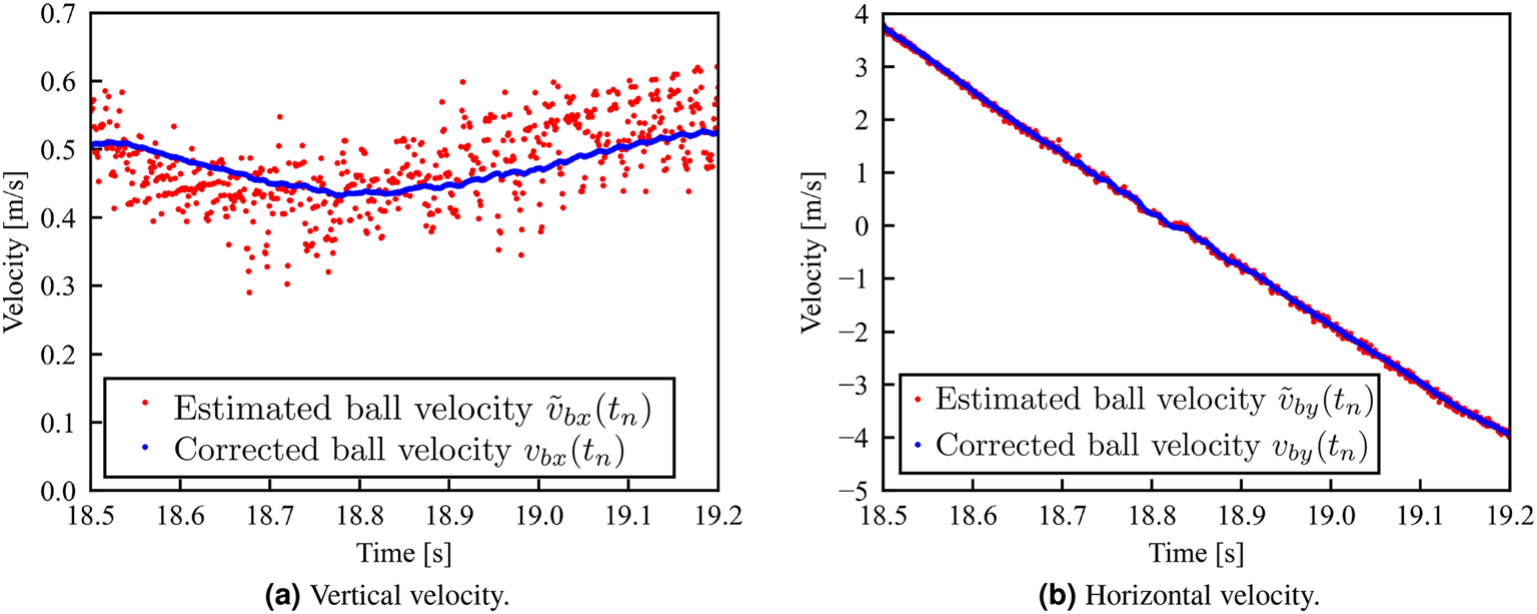
Investigating the effectiveness of corrected ball velocity $\varvec{v_b}$ in Eq. (4) with estimated ball velocity $\varvec{\tilde{v}_b}$ from camera images in Eq. (2).

The position data $\varvec{P_b}(t_n)$ of the ball at time $t_n$ from the start of the measurement were obtained from the filtered image data. Combining this information with 1 ms earlier position information $\varvec{P_b}(t_{n-1})$ , the estimated velocity of the ball calculated from the filtered camera image $\varvec{\tilde{v}_b}(t_n)$ is calculated as follows:

2 $$\begin{aligned} \varvec{\tilde{v}_b} = f \cdot \{\varvec{P_b}(t_n) - \varvec{P_b}(t_{n-1})\}, \end{aligned}$$

where *f* is the sampling frequency at which the image is acquired by the camera; in this paper, it is 1000 fps. Based on the estimated velocity from the camera image, the initial velocity for the velocity calculation in the next frame $\varvec{\bar{v}_b}(t_n) = [\bar{v}_{bx}(t_n), \bar{v}_{by}(t_n)]^T$ is determined as follows:

3 $$\begin{aligned} \left\{ \begin{array}{l} \bar{v}_{bx}(t_{n}) = (1-k_x)v_{bx}(t_n) + k_x \tilde{v}_{bx}(t_n)\\ \bar{v}_{by}(t_{n}) = (1-k_y)v_{by}(t_n) + k_y \tilde{v}_{by}(t_n) \end{array} \right. , \end{aligned}$$

where the correction parameters $k_x$ and $k_y$ were experimentally determined to be 0.01 and 0.15, respectively. Based on the equations of motion, the velocity of the ball at time $t_n$, $v_b=[v_{bx},v_{by}]^T$, is calculated from the one-frame earlier initial velocity as follows:

4 $$\begin{aligned} \left\{ \begin{array}{l} v_{bx}(t_n) = \bar{v}_{bx}(t_{n-1})\\ v_{by}(t_n) = \bar{v}_{by}(t_{n-1}) - g/f \end{array} \right. , \end{aligned}$$

where *g* is the gravitational acceleration. Figure 5a and b plot the corrected velocity of the ball $\varvec{v_b}(t_n)$ and the estimated velocity obtained from the camera image $\varvec{\tilde{v}_b}(t_n)$, respectively. The ideal motion of the system is calculated based on the velocities obtained from Eq. (4).

#### Hitting instruction

**Figure 6:**
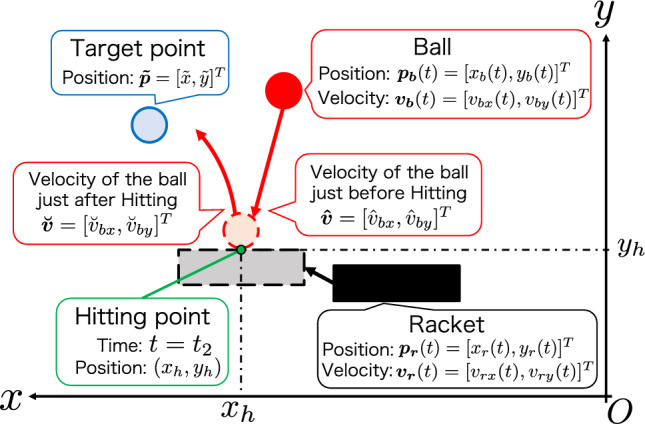
Schematic diagram of the parameters appearing in the hitting instruction algorithm.

Figure 6 provides an overview of the variables used in the hit instructions. In this study, the ball moves in a parabolic motion. For this reason, the ideal racket position is calculated from the equation for parabolic motion. If the height at which the ball is hit is known, it is possible to calculate the time at which it reaches this height. If the height at which the ball is hit is $y_h$, the time $t_2$ at which it reaches that height can be expressed as follows:

5 $$\begin{aligned} t_2 = \frac{v_{by}(t)}{g} + \frac{\sqrt{{\{v_{by}(t)\}^2}+2g(y_{b}(t)-y_h)}}{g} + t, \end{aligned}$$

where the height of the center of mass of the ball at time *t* is $y_b(t)$, and the vertical downward velocity of the ball is $v_{by}(t)$. Time $t_2$ represents the time when the ball is hit back in ideal behavior. Using time $t_2$, the velocity of the ball immediately before hitting $\hat{\varvec{v}}=[\hat{v}_{bx}, \hat{v}_{by}]^T$ is calculated from velocity of ball $\varvec{v_b}(t)$ as follows:

6 $$\begin{aligned} \left\{ \begin{array}{l} \hat{v}_{bx} = v_{bx}(t) \\ \hat{v}_{by} = -g(t_2 - t) + v_{by}(t) \end{array} \right. . \end{aligned}$$

In addition, using the time of hitting $t_2$ and the position and velocity of the ball at time *t*, the horizontal position $x_h$ of hitting the ball back was computed as follows:

7 $$\begin{aligned} x_h = x_b(t) + v_{bx}(t)\cdot (t_2 - t). \end{aligned}$$

In parabolic motion, if the projectile is launched in the vertical direction with an initial velocity of $\breve{v}_{by}$, the time $t_3$ at which the projectile reaches the trajectory tip is represented as follows:

8 $$\begin{aligned} t_3 = \frac{\breve{v}_{by}}{g} + t_2. \end{aligned}$$

As the position of the ball at time $t_3$ coincides with the target point $\varvec{{\tilde{p}}} = [{\tilde{x}}, {\tilde{y}}]^T$, the velocity $\varvec{\breve{v}} = [\breve{v}_{bx}, \breve{v}_{by}]^T$that the ball should have immediately after contact is obtained as follows:

9 $$\begin{aligned} \left\{ \begin{array}{l} \breve{v}_{bx} = ({\tilde{x}} - x_h)\sqrt{\frac{g}{2({\tilde{y}} - y_h)}}\\ \breve{v}_{by} = \sqrt{2g({\tilde{y}} - y_h)} \end{array} \right. . \end{aligned}$$

In this system, the ball is in contact with a frictional racket surface. In this study, the coefficients of repulsion in the vertical and horizontal directions concerning the racket surface were assumed as $e_v$ and $e_h$, respectively, and the repulsion in the horizontal direction concerning the racket surface also holds. Moreover, the velocity of the racket remained unchanged before and after the contact. Therefore, considering the ideal behavior based on the velocity of the racket, $\varvec{v_r}(t) = [v_{rx}(t), v_{ry}(t)]^T$, the velocity $\varvec{v_r}(t_2)$ that the racket should have at the time $t_2$ for the ball to have a velocity $\varvec{\breve{v}}$ immediately after contact is computed as follows:

10 $$\begin{aligned} \left\{ \begin{array}{l} {v}_{rx}(t_2) = \frac{\breve{v}_{bx}-e_{h}\hat{v}_{bx}}{1+e_{h}}\\ {v}_{ry}(t_2) = \frac{\breve{v}_{by}-e_{v}\hat{v}_{by}}{1+e_{v}} \end{array} \right. . \end{aligned}$$

The ideal changes in the velocity of the racket in the proposed algorithm were defined by the cubic spline functions $S_x^h(t)$ and $S_y^h(t)$ concerning time *t*, where the starting point was $(t_1, 0)$ and the ending point was $(t_2, {v}_{rx}(t_2))$ or $(t_2, {v}_{ry}(t_2))$. The cubic spline function provides the ideal velocity of the racket at time *t*, $\varvec{v_r}(t)$, as follows:

11 $$\begin{aligned} \left\{ \begin{array}{l} v_{rx}(t) = S_x^h(t) \\ v_{ry}(t) = S_y^h(t) \end{array} \right. . \end{aligned}$$

Subsequently, using the position of the racket before $\Delta t$ seconds, the position of the racket at time *t*, $\varvec{p_r}(t)=[x_r(t), y_r(t)]^T$, is computed as follows:

12 $$\begin{aligned} \left\{ \begin{array}{l} x_r(t) = v_{rx}(t)\Delta t + x_{r}(t-\Delta t)\\ y_r(t) = -0.5g(\Delta t)^2 + v_{ry}\Delta t + y_r(t - \Delta t) \end{array} \right. . \end{aligned}$$

This formula defines $\varvec{p_r}(t)$ as the ideal racket position at time *t* during the hit instruction.

#### Reaching instruction

The racket was moved vertically and horizontally during the hit instructions. Therefore, the position of the racket must be adjusted in advance such that it crosses position $p_r(t_2)$ at time $t_2$ when it hits the ball back. The total amount of movement of the racket during the hit instruction, $\varvec{\Delta p}=[\Delta x, \Delta y]^T$, was obtained by integrating $\varvec{v_r}(t)$ over time in Eq. (11) as follows:

13 $$\begin{aligned} \varvec{\Delta p} = \int _{t_1}^{t_2}\varvec{v_r}(\tau )d\tau . \end{aligned}$$

Let $\varvec{p_r}(t_0)$ be the position of the racket at time $t_0$ when the predicted vision starts, and let the racket be moved to position $\varvec{\bar{p}_r} = [x_h - \Delta x, y_h - \Delta y]^T$ at time $t_1$ upon reaching the instruction. Therefore, the system computes the ideal trajectory of the racket using the cubic spline functions $S_x^r(t)$ and $S_y^r(t)$ with respect to time *t*, where $(t_0, x_r(t_0))$ is the initial point, $(t_1, x_h - \Delta x)$ is the endpoint, $(t_0, y_r(t_0))$ is the initial point, and $(t_1, y_h - \Delta y)$ is the endpoint, respectively. The position of the racket at time *t*, $\varvec{p_r}(t)$, is expressed as

14 $$\begin{aligned} \left\{ \begin{array}{l} x_r(t) = S_x^r(t) \\ y_r(t) = S_y^r(t) \end{array} \right. . \end{aligned}$$

Here, $\varvec{p_r}(t)$ obtained in this equation is used as the ideal position of the racket at time *t* in the reaching instruction.

#### Calculation method of force output value

**Figure 7:**
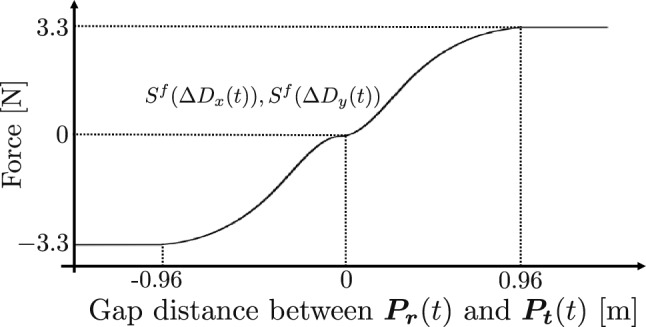
Spline functions for calculating force output values.

The proposed system teaches motions that transcend human motor abilities by providing force feedback directed towards the calculated ideal behavior in response to the manipulation commands of the operator. However, if the guidance is completely idealized, the operator becomes dependent on the instruction and is not considered to promote human motor learning^15^. Therefore, in this study, a force-feedback teaching algorithm was developed such that the operator does not depend entirely on the ideal trajectory. In the proposed system, the guided command value $\varvec{p_t}(t)$ is determined at the internal division point based on the ideal operation command $\varvec{p_i}(t)$ and the human operation command $\varvec{p_r}(t)$ at time *t* as follows:

15 $$\begin{aligned} \varvec{p_t}(t) = (1 - \alpha )\varvec{p_r}(t) + \alpha \varvec{p_i}(t). \end{aligned}$$

where $\alpha$ represents the strength of the assist which determines the guided command value. We have also conducted preliminary experiments to determine which value of alpha is the better. We asked some participants to perform the same task as in this experiment with several different alpha values, and then we conducted a questionnaire to determine which alpha value was better for participants to manipulate and hit back accurately on their own. As a result, we determined to set $\alpha$ to 0.5, such that $\varvec{p_t}(t)$ is the midpoint between $\varvec{p_i}(t)$ and $\varvec{p_r}(t)$. The force output value is defined in a nonliar manner based on $\Delta \varvec{D}(t) = [\Delta D_x(t), \Delta D_y(t)]^T$, the deviation between the target point $\varvec{p_i}(t)$, and the operator’s operation command $\varvec{p_r}(t)$. It was calculated using cubic spline functions $S^{f}(\Delta D_x(t))$ and $S^{f}(\Delta D_y(t))$ with both the initial point at (0, 0) and the end point at (0.96, 3.3) . Furthermore, an endpoint value of 0.96 was the gap distance between the real racket position $\varvec{P_r}(t)$, and the target racket position $\varvec{P_t}(t)$ meters with setting experimentally, and 3.3 was the highest output value of the device. The force direction can also be defined by extending the spline function to negative distances. Figure 7 shows the function and the equation for the output force value $\varvec{f}(t) = [f_x(t), f_y(t)]^T$ is as follows:

16 $$\begin{aligned} \left\{ \begin{array}{l} f_x(t) = S^{f}(\Delta D_x(t)) \\ f_y(t) = S^{f}(\Delta D_y(t)) \end{array} \right. . \end{aligned}$$

## Results

**Figure 8:**
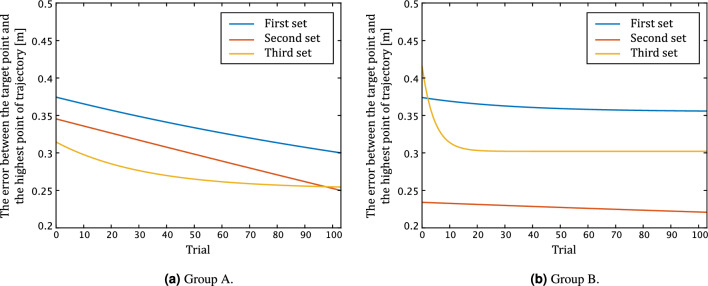
The learning curve for each group. This is the result of the approximate with Eq. (1).

Figure 8 shows the learning curve for the subjects belonging to each group. They were computed by applying the exponential approximation shown in Eq. (1) with the mean errors obtained by the goal-directed task for each group on a trial-by-trial basis. Figure 8a shows the learning curves for Group A, and Fig. 8b shows the learning curve for Group B. The blue, red, and yellow lines are the results for the first, second, and third sets, respectively.

Group A was the control group that did not use the predictive-vision system in any of the three sets. The error in Group A decreased as the number of sets increased from the first to the third. Furthermore, the error in each set decreased as the trials proceeded. The result indicated that Group A gradually progressed in motor adaptation over the 312 trials. In contrast, Group B was assisted by the predictive-vision system only in the second set and did not use the system in the remaining sets. In the first set of trials, Group B showed the same tendency as Group A. In the second set, the performance in the first trial was equivalent to that in the final trial of the third set in Group A, suggesting that the use of the predictive-vision system improved motor performance. This improved performance was maintained throughout the second session in Group B. In the third set, the error rebounded immediately after the washout of the predictive-vision system but rapidly decreased over 10 trials. These results suggest that the subject could adapt to the goal-directed task without the predictive-vision system.

Table 1 gives the performance improvement in terms of mean errors for each subject. The values were calculated by subtracting the first set value from the second or third set value, respectively. We conducted a two-way factorial ANOVA on the mean values. The results indicate an F-value of 4.07 and a p-value of $5.74 \times 10^{-2}$ for the main factor test regarding the between-sets. The test for the main factor between groups resulted in an F-value of 3.78 and a p-value of $6.60 \times 10^{-2}$, indicating no difference in the main factor. Nevertheless, the p-value for the interaction was $1.69 \times 10^{-2}$, confirming the interaction. To compare how much performance improved from initial performance between subject groups and between sets, multiple comparisons using Tukey’s HSD method were conducted. The result shows there was a difference between the performance improvement in the second set of Group B and the performance improvement in the second set of Group A(p-value = $1.85 \times 10^{-2}$). Additionally, there was a difference between the performance improvement in the third set of Group A and the second set of Group B (p-value = $4.98 \times 10^{-2}$). These results confirm that the predictive-vision system enhances the performance improvement from the baseline performance. Conversely, the performance improvement of Group B in the third set was tended to be worse than that of Group A in the third set (p-value = 0.974).

**Figure 9:**
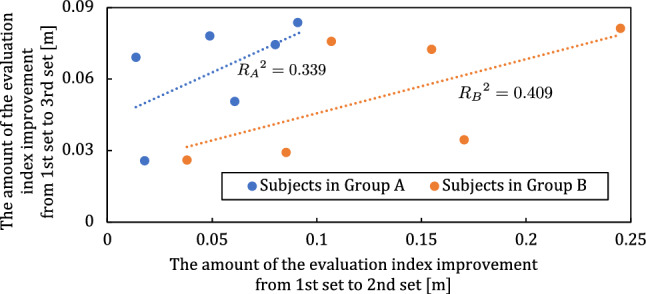
Correlation between the performance improvement from the first set to the second set and improvement from the first set to the third set in the task of this experiment.

Figure 9 shows the correlation of the improvement in the error for each group. The blue and orange data points indicate the amount of error improvement for Groups A and B, respectively. The horizontal axis represents the improvement from the first set to the second set, and the vertical axis represents the improvement from the first set to the third set. These improvements are calculated by subtracting the average error of the first set from the average error of each set. The graph on correlation shows a tendency for each group to have a proportional relationship with the amount of error. In addition, the horizontal axis tended to be higher in Group B, which used the predictive-vision system.

**Table 1:** The performance improvement on mean error from the first set to the second set and from the first set to the third set. This is calculated by subtracting the value of the error obtained for each set from the value of the first set.

(a) Group A.		
Subject	From the first set to the second set	From the first set to the third set
A	0.0135	0.0691
B	0.0909	0.0838
C	0.0608	0.0507
D	0.0489	0.0782
E	0.0802	0.0744
F	0.0178	0.0258
All data	0.0520	0.0637

(b) Group B.		
Subject	From the first set to the second set	From the first set to the third set
G	0.0853	0.0293
H	0.170	0.0346
I	0.155	0.0725
J	0.244	0.0813
K	0.0379	0.0261
L	0.107	0.0758
All data	0.133	0.0533

## Discussions

In this study, we measured the errors obtained from the upper limb reaching and ball-hitting tasks in Group A (control group) and Group B (predictive-vision group) to investigate the usefulness of the predictive-vision system. The results show that the errors in the goal-directed task was improved in the second set of tasks using the predictive-vision system in Group B than in Group A (indicated by red lines in Fig. 8). This result suggests that the predictive-vision system improves the accuracy of the hand position motion in a goal-directed task. In addition, when comparing the improvement in the motor error between the first and second sets for each group, Group B showed greater improvement than Group A (indecated in Table 1 and in Fig. 8), further supporting the usefulness of the predictive-vision system. Several studies have shown that haptic guidance by robotic systems can promote motor learning^2,3^. However, such guidance is limited to those with pre-defined motion trajectories or assist forces, regardless of individual differences in motion. Conversely, the proposed predictive-vision system can sequentially calculate the guidance motion by real-time updating the command parameters based on motion data while simultaneously performing the hitting motion. The system is novel because guidance motion trajectories and force vectors vary from person to person. We consider that the ability to appropriately estimate the operator’s movement intention and modify the assistance has led to a reduction in hand-positioning errors that occur in goal-directed reaching tasks. Furthermore, the proposed predictive-vision system has a support ratio of 50%, which means that humans do not entrust all their power to the support system. Therefore, we believe that the proposed support algorithm is valuable because it can improve motor performance without interfering with personal performance power.

Second, the correlations between the error differences between the first and second sets and between the error differences between the first and third sets were higher in Group B than in Group A. This result suggests that improvements in the predictive-vision system would lead to higher learning rates. Many physiological studies on motor learning have focused on the importance of sensory modalities such as vision and proprioception in online motor control^16–18^. For example, training a hand with rotational visual feedback updates these predictions, which contribute to motor control^19^. Moreover, the sensation of hand position (i.e., intrinsic sensation) based on afferent pathways from the muscles to the brain is also effective in readjusting motor control^19^. Considering these previous studies, using the predictive-vision system to update new and more appropriately modified eigen-sensory and visual information may lead to an improvement in motor performance (i.e., facilitates motor learning) compared to the case in which the predictive-vision system is not used. On the other hand, it should be noted that even in group B, there was variation in the amount of error improvement by the predictive-vision system, with a division between three individuals with a large improvement in motor performance between one set and three sets and three individuals with a smaller improvement in comparison (shown in Fig. 9). These results are attributed to the reliability of the proposed system. For example, even if the predictive-vision system assists the same force vector, it’s up to the individual how he/she relates the force exerted by him/herself to it. To further verify this suggestion, it is necessary to extract the relationship between the force vector output by the system and the user’s own force vector, for example, by investigating the similarity between these two vectors. It is crucial to estimate each user’s utilization strategy for the predictive-vision system through these verifications in the future.

Moreover, when the predictive-vision system is removed, the motion error rebounds (returns to the base), and the improvement effect cannot be sustained (indicated by the red and yellow lines in Fig. 8b). The rebound of the motion error by removing the external force applied during motion is consistent with the findings of previous studies on motion learning^16–19^. We consider the predictive-vision system to be valuable because it can examine motor adaptation to more time-constrained dynamic movements with accuracy comparable to that of previously studied goal-directed reaching tasks. It is possible to validate new knowledge about human motor learning and motor adaptation to more time-constrained movements by manipulating the auxiliary parameters of the predictive-vision system. Some studies on teleoperation training focused on assistance modifications based on operator skills^20–22^. For example, it has been suggested that adjusting the control level according to the operator skill evaluation using machine learning effectively improves the user experience^20^. It has also been reported that when a single subject is provided with the assistance of a gradually decreasing intensity, high performance during assist is maintained, even when the assist is completely removed^21^. In the future, it may be possible to optimize the assistance algorithm of the proposed system and develop a system that can promote motor learning by adding experiments in which the percentage of assistance is not limited to 50% but is set in several steps.

## Conclusions

In this study, we proposed a system that provides motion force feedback to human hand positions in time-constrained, goal-directed tasks. The proposed system is equipped with a predictive-vision system that uses high-speed image measurement technology to estimate the state quantities of the objects observed by the camera in the near future. Based on the estimated future state quantities, real-time force feedback is achieved in a time-constrained environment.

Experiments with 12 healthy subjects were conducted to investigate the effects of the proposed system on motion performance in a time-constrained goal-directed reaching and ball-hitting task. In the experiment, the subjects were divided into two groups, a control group, and a predictive-vision group, and the motion feedback by the system was only provided in the middle phase of the experiment for the predictive-vision group. The motion feedback was removed in the other task of the experiment, including the final phase of the predictive-vision group. The results showed that the predictive-vision group tended to perform better during the motion feedback, even compared to the final phase of the control group. This result suggests that the real-time and force-motion feedback by the proposed system functioned effectively to improve the operator’s motor performance even in a time-constrained task. Focusing on the improvement in motor accuracy at the end of the experiment, a proportional relationship tendency was found between the improvement and the improvement caused by the motion feedback. These results suggest that motor performance may be improved by using the proposed system and modifying the proprioceptive sensation appropriately. However, even in the predictive-vision group, there was variation in the improvement of motor accuracy. In the future, it is anticipated to estimate the cause of the variation in the improvement of motor accuracy by estimating the utilization strategy of each operator’s motion feedback. Finally, when the motion feedback by the proposed system was removed, the motor error of the subjects rebounded, and the improvement effect was not sustained. This is consistent with the findings of previous studies on motor learning. Some of these studies have modified the motion assist as the training progressed. In the future, optimizing the assist algorithm like these studies could lead to the development of a system that improves motor learning.

## Data Availability

All the datasets generated in this study are available from the corresponding authors.
